# Disrupted neurovascular-endocrine coupling in type 1 diabetes with impaired awareness of hypoglycemia

**DOI:** 10.1172/JCI199725

**Published:** 2026-02-19

**Authors:** Pavel Filip, Antonietta Canna, Heidi Grohn, Amir A. Moheet, Anjali F. Kumar, Xiufeng Li, Yuan Zhang, Lynn E. Eberly, Elizabeth R. Seaquist, Silvia Mangia

**Affiliations:** 1 Center for Magnetic Resonance Research, University of Minnesota, Minneapolis, Minnesota, USA.; 2 Department of Neurology, Charles University, First Faculty of Medicine and General University Hospital, Prague, Czech Republic.; 3 Department of Cybernetics, Czech Technical University in Prague, Prague, Czech Republic.; 4 Kuopio University Hospital, Diagnostic Imaging Center, Kuopio, Finland.; 5 Department of Medicine and; 6 Division of Biostatistics and Health Data Science, University of Minnesota, Minneapolis, Minnesota, USA

**Keywords:** Endocrinology, Neuroscience, Diabetes, Diagnostic imaging

## Abstract

**BACKGROUND:**

Recurrent hypoglycemia in type 1 diabetes (T1D) may culminate in impaired awareness of hypoglycemia (IAH). While neuroimaging studies identified affected brain regions, more complex perspectives integrating vascular dynamics with endocrine profile are needed.

**METHODS:**

Here, 26 healthy adults, 30 T1D patients with normal hypoglycemia awareness (NAH), and 25 T1D patients with IAH underwent a hyperinsulinemic stepped clamp (euglycemia → hypoglycemia 50 mg/dL) combined with pseudo-continuous arterial spin-labeling MRI. Cerebral blood flow (CBF) and sympathetic vasomotor range (0.02–0.05 Hz) CBF oscillations were modeled against serially sampled plasma cortisol, epinephrine, norepinephrine, and glucagon.

**RESULTS:**

In healthy individuals treated as controls, hypoglycemia evoked robust thalamo-striatal and salience–interoceptive CBF increases (mean Cohen’s *d* across significant clusters = 0.93) and suppression of vasomotor oscillations (*d* = 0.71). T1D retained CBF response but failed to attenuate oscillations (*d_T1D>controls_* = 0.43). IAH further blunted hypoglycemia-associated CBF increase, especially in thalamus, striatum, and insula (*d_NAH>IAH_* = 0.51). Hormone-CBF coupling differed quantitatively: cortisol/epinephrine–CBF correlations were positive in controls (*r* = 0.37/0.26), negative in NAH (–0.16/–0.40), and strongly positive in IAH (0.42/0.46).

**CONCLUSION:**

Thus, our findings indicate that T1D disrupts dynamic, sympathetic modulation of CBF, whereas IAH additionally impairs perfusion reserve and shows maladaptive catecholamine-dependent CBF regulation, suggesting a qualitatively distinct neurovascular phenotype.

**TRIAL REGISTRATION:**

ClinicalTrials.gov: NCT02747680 and NCT02866435.

**FUNDING:**

NIH (P41-EB-015894, P30-NS-076408, R01-DK-099137, R56-DK-099137, and DP1 AG093028); National Center for Advancing Translational Sciences of the NIH (KL2-TR-000113 and UL1-TR-000114); DP1 AG093028; Charles University, Czech Republic (Cooperatio Program, research area NEUR), Brain Dynamics (grant number CZ.02.01.01/00/22_008/0004643); General University Hospital in Prague (MH CZ-DRO-VFN64165).

## Abstract 2

Type 1 diabetes alters brain blood-flow and hormone responses to low blood sugar, helping explain dangerous loss of hypoglycaemia awareness.

## Introduction

The human brain relies almost exclusively on a continuous glucose supply from the bloodstream; its capacity to utilize alternative substrates is limited, yet its metabolic demands are high and endogenous energy reserves are restricted (1). Levels 2 and 3 hypoglycemia (HG) — defined as blood glucose (BG) of concentrations below 54 mg/dL (3.0 mmol/L) or a severe HG episode necessitating external assistance for recovery due to impaired cognitive function (2) — therefore constitute an acute threat to cerebral energetic stability with potentially severe consequences including seizures, coma, or even death if uncorrected.

To mitigate these dangers, the organism mobilizes a multilayered regulatory cascade operating across several timescales and involving many diverse, often partially redundant, mechanisms. Recent findings suggest that the initial response to declining glycemia may comprise metabolic hyperpolarization of vascular smooth muscle cells and pericytes in the brain (3, 4) coupled with astrocytic release of potent mediators (5), which ensures rapid vasodilation. This is followed by the activation of a distributed autonomic network centered on glucose-sensing neurons within the ventromedial and lateral hypothalamus, the paraventricular nucleus, and several nodes of the ascending reticular activating system (ARAS) (6, 7). Such a complex response then provides systemic hemodynamic support while also modulating regional cerebral blood flow (CBF) patterns to shield metabolically vulnerable territories. Furthermore, it may also be one of the initiators of the counterregulatory hormonal and symptomatic responses necessary for glycemic recovery (8). Concurrently, the systemic surge in circulating epinephrine not only contributes to the development of HG symptoms, but it also modulates both hepatic glucose output and suppresses peripheral glucose uptake, thereby elevating systemic glycemia. Nonetheless, this sympathoadrenal response is generally considered to have a limited impact on overall cerebral parenchymal perfusion (9, 10).

Failure of these physiological safeguards — a scenario not uncommon in type 1 diabetes (T1D) mellitus after recurrent HG episodes — gives rise to impaired awareness of hypoglycemia (IAH). IAH is characterized by a shift of the counterregulatory and symptom awareness thresholds to lower glucose levels together with a reduction in the overall magnitude of those responses. One of its major symptomatic consequences is the diminished ability to perceive the development of decreasing BG levels. IAH T1D patients fail to experience the usual warning symptoms of HG and may remain unaware until neuroglycopenia manifests as confusion or leads to the loss of consciousness (11). Affected patients with IAH lose the typical thalamo-cortical CBF increase seen during HG (12, 13), yet their global CBF may be retained or even exaggerated, with potential regional redistribution. Orbitofrontal and dorsolateral prefrontal hyperemia have been observed only in nondiabetic subjects or T1D patients with normal awareness of hypoglycemia (NAH), while hippocampal hypoperfusion has been reported in both NAH and IAH (14, 15). Furthermore, functional MRI has revealed absent recruitment of cortico-striatal and frontoparietal networks in patients with IAH (16).

Collectively though, the referenced literature remains fragmented, occasionally contradictory, and frequently underpowered — often reduced to statements on null within-group findings rather than statistically more rigorous group-by-condition effects. Larger cohorts, particularly of patients with IAH, with joint modeling of CBF and systemic hormonal responses to HG, are notably absent, thereby constraining further elucidation of cerebrovascular adaptation specific to IAH.

In this context, the present study pursued 3 main objectives: (a) to characterize the canonical cerebral perfusion response to acute HG and its physiological coupling with counterregulatory hormone release, (b) to determine the manner in which T1D and IAH distort these neurovascular responses, and (c) to establish the manner in which T1D and IAH distort the hormone-neurovascular response coupling. We approached the analysis in a hierarchical manner, first characterizing the broader alteration present in T1D in general and only then identifying additional deviations specific to IAH within that pathological context. This framework was designed to evaluate whether IAH represents merely a quantitative continuum of T1D pathology or a qualitatively distinct pattern. To provide a more nuanced understanding, we interrogated advanced CBF estimation methodologies with cortical surface reconstructions alongside quantified low-frequency (0.02–0.05 Hz) autonomic CBF fluctuations, a putative proxy of regional sympathetic vasomotor tone (17–19).

## Results

Basic demographic and clinical information is provided in Table 1; the enrollment and exclusion process is summarized in Supplemental Figure 2 (supplemental material available online with this article; https://doi.org/10.1172/JCI199725DS1). Ultimately, 26 patients acting as healthy controls (HCs) and 55 patients with T1D (30 NAH and 25 IAH) were included in the final analysis. Most exclusions were necessitated by incomplete datasets or second pseudo-continuous arterial spin-labeling (PCASL) MRI protocol incompatibility, which exhibited 2 orders of magnitude larger robust Mahalanobis distance (median *D*^2^ ≈ 525) than the other 2 acquisition protocols (median *D*^2^ ≈ 5.5). While HCs (32.1 ± 11.8 years; 12 female/14 male patients) with physiological glycated hemoglobin (HbA1c) values (5.2% ± 0.3 %) did not differ substantially from the aggregated T1D cohort (35.7 ± 13.0 years; 32 female/23 male patients), NAH patients were significantly younger compared with IAH patients (29.6 ± 9.0 years vs. 43.0 ± 13.4 years; *P* < 0.001), with shorter disease duration (15.8 ± 7.5 vs. 26.1 ± 13.4 years, *P* = 0.002) and lower Clarke (1.1 ± 1.0 vs. 4.4 ± 1.2, *P* < 0.001) and Gold scores (2.5 ± 1.0 vs. 4.4 ± 1.3, *P* < 0.001). There were no statistically significant differences in BG control based on glycated hemoglobin levels between IAH and NAH. Clamp-related BG development over the course of the experiment is depicted in Supplemental Figure 3.

### Physiological counterregulatory response.

**Figure 1:**
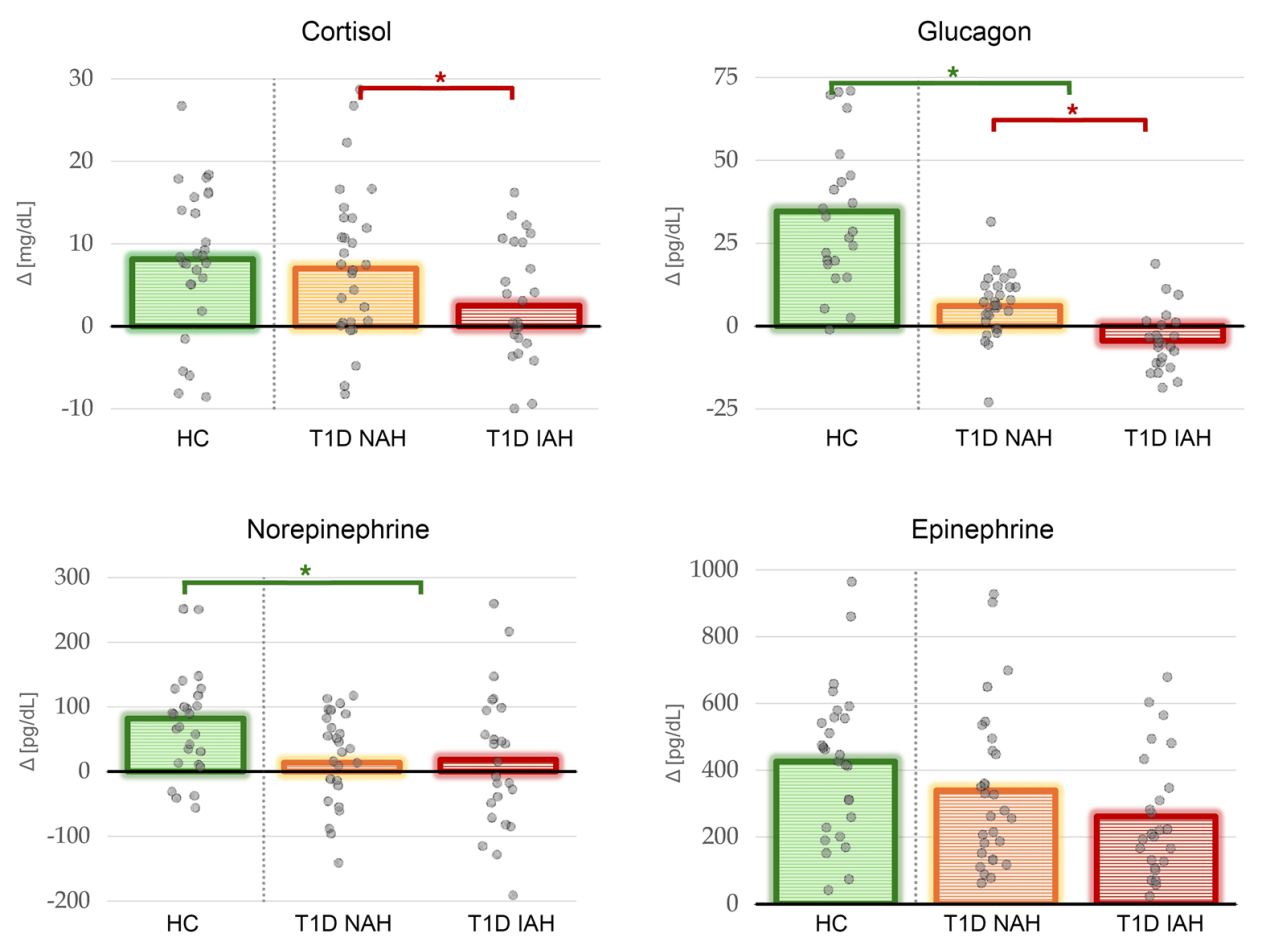
Hormonal response to HG. Bar heights above 0 represent subgroup averages, calculated as the average of the last 2 HG measurements minus the average of the 2 baseline measurements (5 samples in total; the initial hypoglycemic value was excluded to obtain a steadier estimate for this simplified visualization). Individual participant values are depicted as gray circles, with jitter along the *x* axis for better visibility. Significant intergroup differences between HCs and pooled T1D patients based on linear mixed-effect models (see Table 2) are marked with green bar and asterisk; significant differences between IAH and NAH T1D subgroups are marked with red bar and asterisk (*P_FDR_ < 0.05). See Table 2 for formal statistical evaluation considering full time course of hormonal response.

Among HCs, linear mixed-effects modeling (LMEM) revealed significant HG-associated hormonal level linear increase over time for cortisol, epinephrine, glucagon, and norepinephrine alike (*P*_FDR_ < 0.001). See Table 2 and Figure 1 for further information.

### Cerebral hemodynamic response to acute HG and its coupling with hormonal response in HCs.

**Figure 2:**
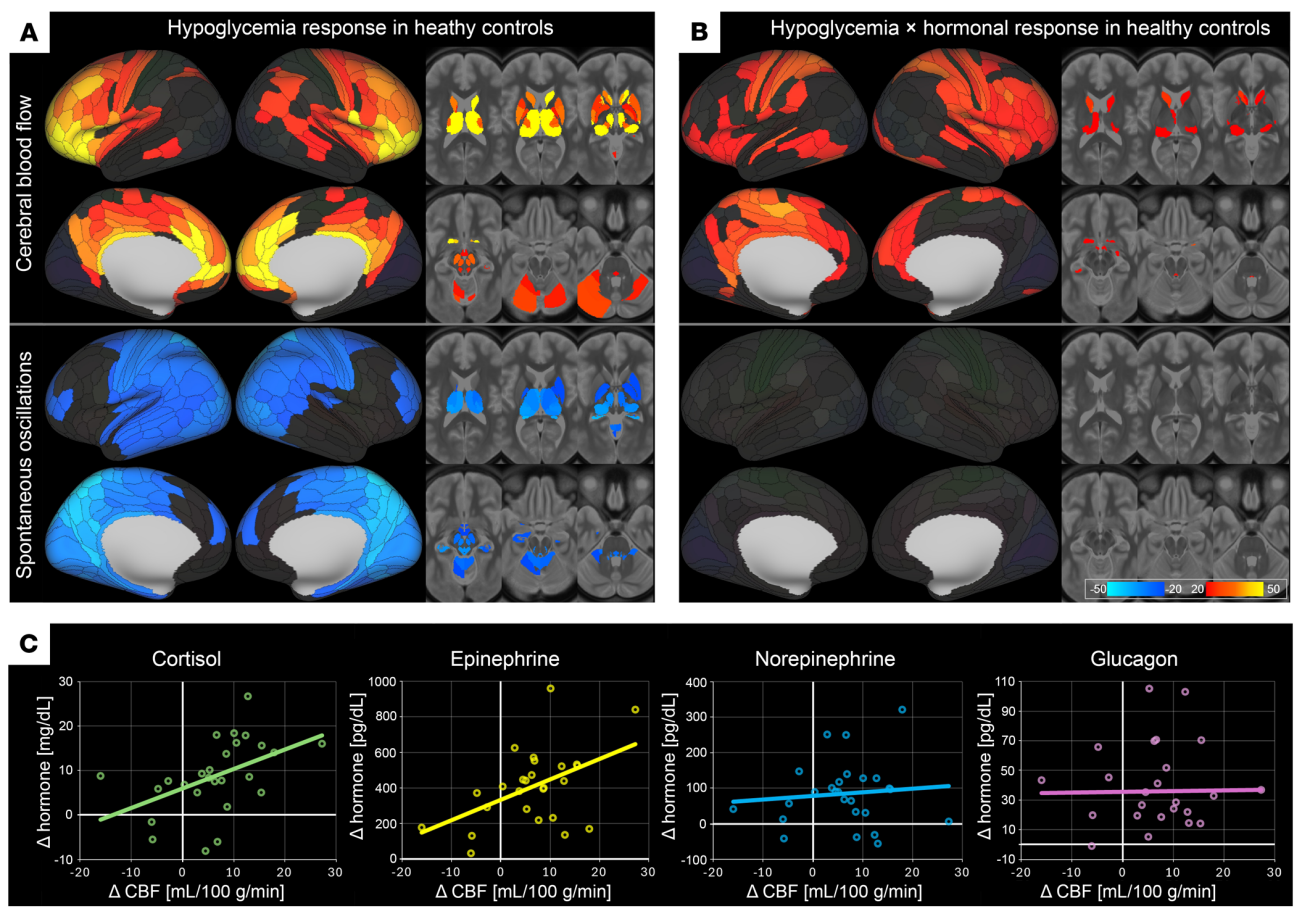
Cerebral hemodynamic response to HG in HCs. (**A**) HG > EU contrast for CBF and spontaneous CBF oscillations. (**B**) Interaction between HG and composite hormonal response (cortisol, epinephrine, norepinephrine, and glucagon) by omnibus *F* test. Maps are provided as *z* statistics with threshold-free cluster enhancement, displayed at *P* < 0.05 (FDR corrected jointly across parcellation units, modalities, and contrasts). Warm colors (yellow–red) indicate significant increase; cool colors (blue) denote the reverse effect. Subcortical structures illustrated on 6 axial slices (MNI *z* = 15, 6, –3, –12, –21, and –30). Images follow neurological convention (right = right). (**C**) Scatterplots depicting HG-induced responses in absolute units of cortical CBF (*x* axis) versus individual hormones (*y* axis) for each hormone separately, with superimposed linear trend lines for visualization. For detailed cluster statistics and anatomical labels, see Supplemental Tables 1 and 2. For comparison of CBF versus hormonal response relationship in IAH, see Figure 4.

HG produced a marked CBF increase (mean effect size [Cohen’s *d*] across all clusters 0.927) within the bilateral salience–interoceptive network encompassing the anterior and mid-cingulate cortices, anterior insula, and dorsolateral prefrontal cortex (DLPFC), extending over the premotor and inferior parietal cortex (Figure 2A and Supplemental Table 1). Subcortically, a robust rise in CBF within thalamus, striatum, and pallidum was accompanied by posterior hypothalamus and mesencephalic reticular formation. Contrary to perfusion, low-frequency CBF fluctuations were suppressed during HG throughout the same salience–interoceptive circuit, but also brainstem monoaminergic nuclei, thalamus, and hippocampus (mean *d* = 0.713).

The magnitude of the integrated hormonal response (omnibus *F* test) was associated with CBF increase in a limbic–fronto–striatal axis (Figure 2B and Supplemental Table 2), specifically anterior/posterior cingulate, insula, DLPFC, amygdala, ventral striatum, and anterior-inferior hypothalamus. Cortisol and epinephrine (Figure 2C and see below) were the primary drivers of these findings.

### Effect of T1D on counterregulatory response, CBF, and vasomotor fluctuations.

Multivariate comparison of the overall hormonal response between HCs and the pooled T1D group confirmed significant overall intergroup differences (*F* = 7.716, *P*_FDR_ = 0.003), driven by significantly attenuated slope for glucagon (*P*_FDR_ < 0.001) and norepinephrine (*P*_FDR_ = 0.001), whereas no significant differences were observed for cortisol (*P*_FDR_ = 0.133) or epinephrine (*P*_FDR_ = 0.109) (Table 1).

**Figure 3:**
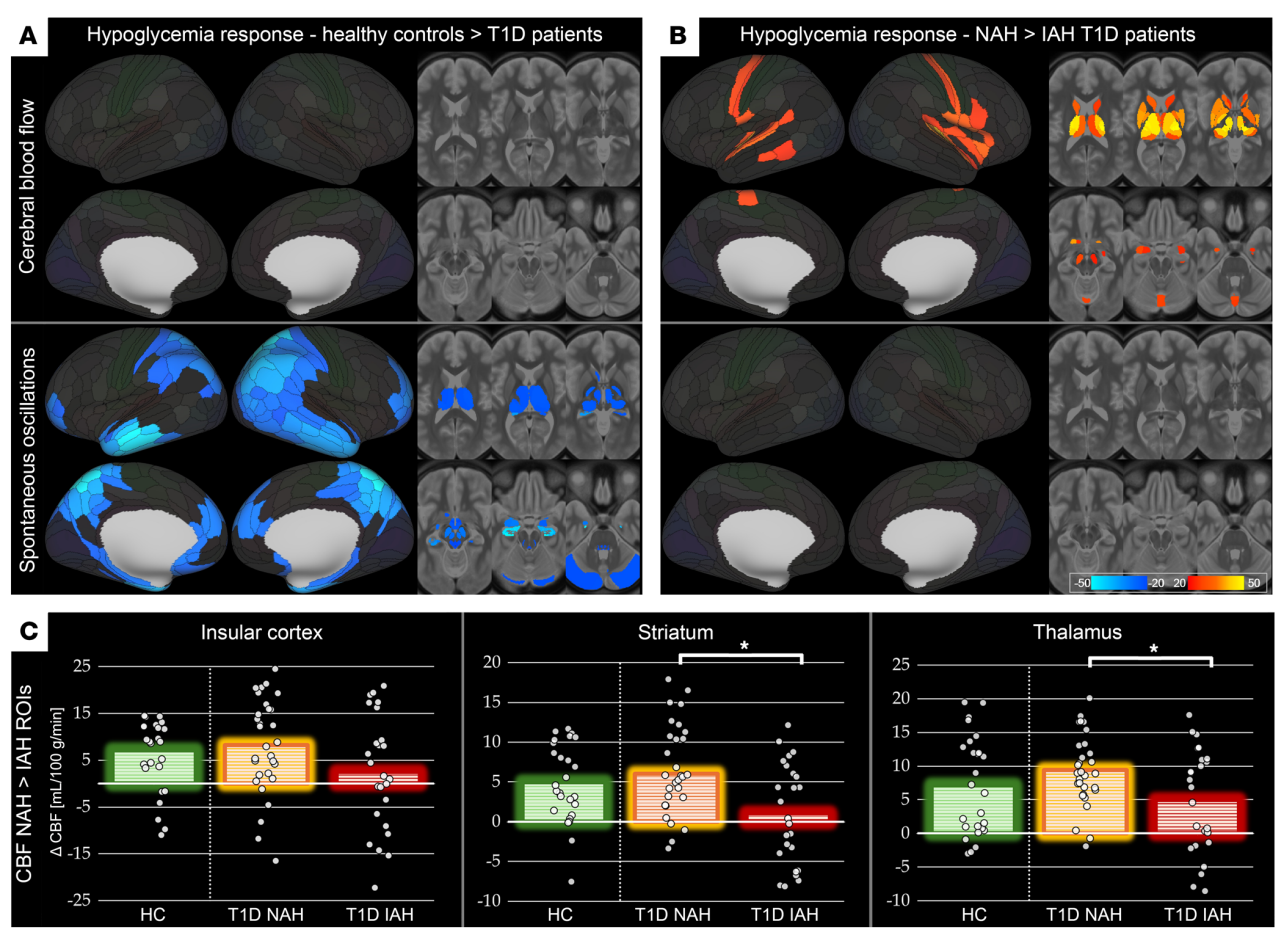
Group contrasts for cerebral hemodynamic response to HG. (**A**) HCs versus pooled cohort of T1D patients. (**B**) T1D patients with NAH versus IAH. Each panel shows maps of *z* statistic with threshold-free cluster enhancement for CBF and spontaneous CBF oscillations, displayed at *P* < 0.05 (FDR corrected jointly across parcellation units, modalities, and contrasts). Warm colors (yellow–red) denote a greater response in the first group; cool colors denote the reverse effect. Subcortical effects illustrated on 6 axial slices (MNI *z* = 15, 6, –3, –12, –21, and –30). Images follow neurological convention (right = right). (**C**) Regional CBF response (absolute difference in mL/100 g/min) in 3 regions of interest derived from contrast B (insular cortex, striatum, and thalamus). White circles represent individual participants (jittered along the *x* axis for clarity); bar heights above 0 represent group means for HCs, T1D patients with NAH, and T1D patients with IAH; asterisks mark statistically significant differences as the basis for the selection of regions of interest (*P_FDR_ < 0.05). For detailed cluster statistics and anatomical labels, see Supplemental Tables 3 and 4. ROI, region of interest.

**Figure 4:**
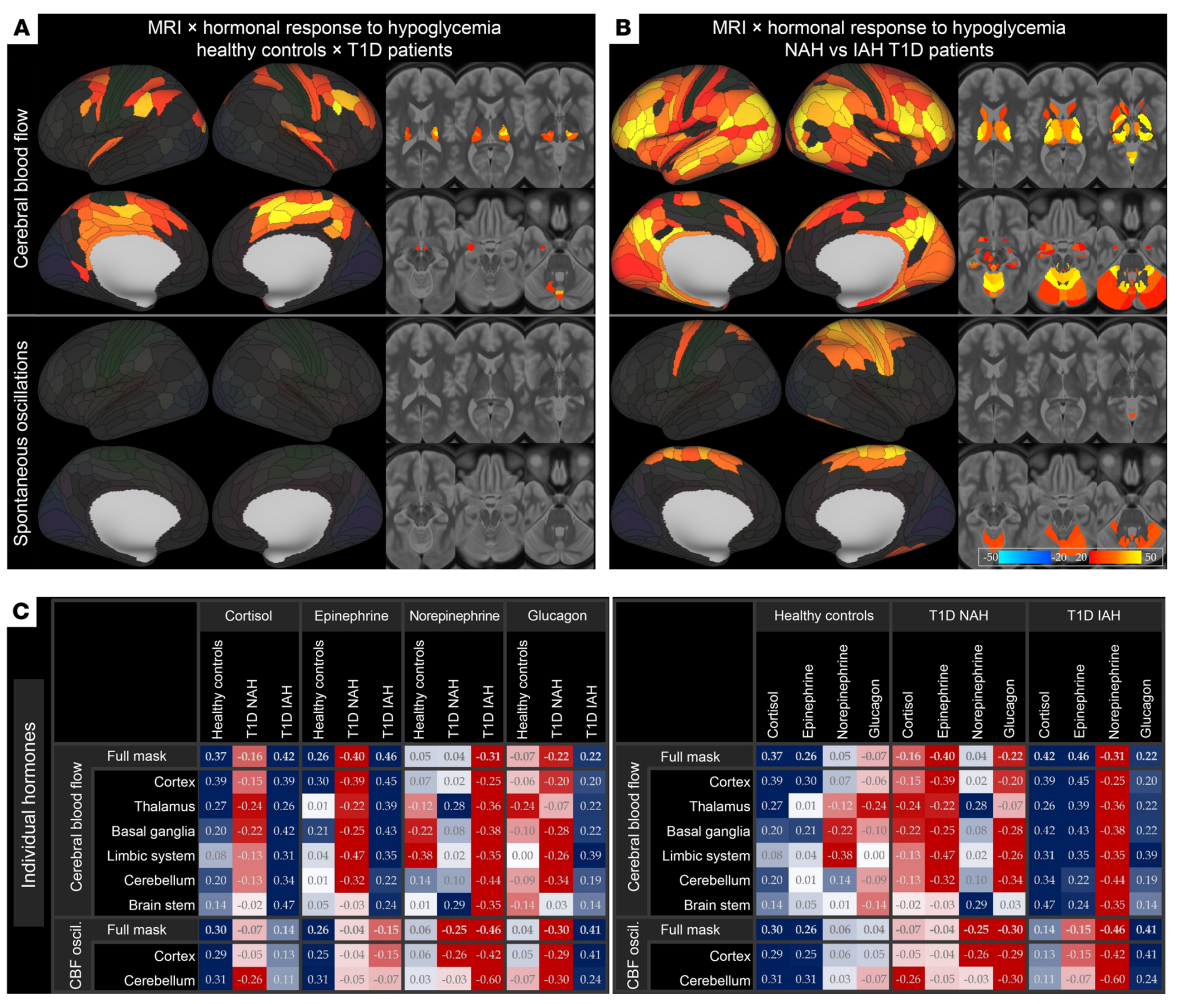
Group differences in HG-elicited hormone-CBF coupling. (**A**) HCs versus pooled cohort of T1D patients. (**B**) T1D patients with NAH versus IAH. Each panel shows maps of *z* statistics with threshold-free cluster enhancement for CBF and spontaneous CBF oscillations, modeling the interaction between group and the composite hormonal response. Maps are thresholded at *P* < 0.05 (FDR corrected jointly across parcellation units, modalities, and contrasts). Subcortical effects illustrated on 6 axial slices (MNI *z* = 15, 6, –3, –12, –21, and –30). Images follow neurological convention (right = right). (**C**) Heatmap matrices (Pearson’s *r*) summarizing hormone-brain response correlation separately for all 4 hormones considered and 3 subject groups in aggregated regions of interest derived from **B**. Red hues denote negative and blue hues positive coupling coefficients. Hormone (left) and subject group (right side) column aggregation provided separately for better concept visualization. For detailed cluster statistics and anatomical labels, see Supplemental Tables 5 and 6.

Moreover, T1D patients failed to show the HG-induced CBF fluctuation suppression detected in HCs, exhibiting differences from HCs in the hippocampus, posterior cingulate, parietal association cortex, insula, and basal ganglia, as well as within the hypothalamus and pontine reticular complex (mean *d* = 0.430; Figure 3A and Supplemental Table 3). T1D also displayed divergent hormone-dependent CBF modulation in a bilateral, but right side–dominant, cingulate–insula–DLPFC network, bilateral thalami, intermediate hypothalamus, and cerebellar midline (Figure 4A and Supplemental Table 5).

### Effect of IAH on counterregulatory response, CBF, and vasomotor fluctuations.

Multivariate analysis confirmed significantly different global hormonal response profiles between NAH and IAH (*F* = 6.677, *P*_FDR_ = 0.008). Specifically, glucagon response was significantly lower in patients with IAH (*P*_FDR_ < 0.001), with more modest intergroup difference in cortisol (*P*_FDR_ = 0.0113). No significant differences were detected for epinephrine (*P*_FDR_ = 0.293) or norepinephrine (*P*_FDR_ = 0.862). Counterregulatory symptom scores failed to reveal any significant intergroup differences (Table 1 and Supplemental Figure 4).

IAH led to blunted HG-evoked CBF rise when compared with NAH in the bilateral thalami, pallidum, dorsal striatum, and insula, as well as in sensorimotor cortices and cerebellar vermis (mean *d* = 0.506; Figure 3, B and C, and Supplemental Table 4). By contrast, no statistically significant differences in CBF fluctuations were detected in the IAH versus NAH comparison.

Supplemental 3-group, 1-way ANOVA showed similar regional patterns (Supplemental Figure 5). NAH T1D patients exhibited consistently closer HG CBF response profiles to HCs, out of 14 significant clusters for NAH versus IAH general linear models (GLMs). NAH patients were closer to HCs in 12 clusters, with a mean absolute NAH-HC gap of 1.36 compared with 3.94 mL/100 g/min in IAH.

Furthermore, IAH affected endocrine coupling differences for both CBF and its fluctuations, additionally encompassing diffuse prefrontal, parietal, and visual cortex territories, combined with striato-pallidal complex, amygdala, hippocampus, ARAS, and cerebellar cortex (Figure 4B, Supplemental Table 6).

In the post hoc analysis (Figure 4C), HCs exhibited modest positive coupling between CBF and its fluctuations with both cortisol and epinephrine secretory response, most pronounced in the cortex and basal ganglia, whereas both norepinephrine and glucagon were largely neutral or borderline negative. In NAH T1D, this pattern was actually inverted: cortisol and epinephrine correlations became negative (*r* = –0.16 and –0.40, respectively, for the full mask). This sign reversal trend held true across cortex, thalamus, and the basal ganglia. Norepinephrine associations remained neutral for absolute CBF response but turned negative for CBF fluctuations. Finally, IAH T1D showed exaggerated positive CBF coupling compared with HCs for both cortisol and epinephrine (*r* = 0.42 and 0.46 for the full mask, respectively), holding true for all the regions considered. Norepinephrine correlations were uniformly negative for both CBF and its fluctuations, whereas glucagon exhibited positive coupling with CBF and its fluctuations.

## Discussion

Our investigation provides a comprehensive characterization of cerebral hemodynamic and counterregulatory response to acute HG and its endocrine control in health, T1D, and IAH. Using one of the largest, clamp-controlled cohorts published, we confirmed a robust rise in mean CBF across the thalamus, basal ganglia, and salience–interoceptive network, scaling positively with the integrated counterregulatory hormone response, accompanied by synchronous suppression of very-low-frequency (0.02–0.05 Hz) vasomotor oscillations. T1D was associated with failure to attenuate these vasomotor fluctuations, and in IAH, HG-associated CBF increase was further blunted, especially in thalamo-striatal regions and insular cortex. Most importantly, we identified a qualitative alteration in endocrine-CBF coupling. In individuals with NAH, surges in cortisol and epinephrine were associated with CBF reductions, suggesting an inverted coupling pattern relative to HCs. By contrast, in patients with IAH, elevations in these hormones elicited an exaggerated hyperemic response, signifying qualitatively distinct pathophysiology of IAH superimposed on a T1D background.

### Characteristics of physiological response to HG.

The robust increase in mean CBF documented here in HCs has been hypothesized as the brain’s primary safeguard against energetic failure, mobilizing multiple vascular (5) and autonomic pathways (8). This response (Figure 2) spreads not only to regions detecting declining glucose levels and initiating counterregulatory responses as hypothalamus and ARAS (7, 20), but also to networks mediating conscious recognition and behavioral responses to HG, such as insula and anterior cingulate (21). In parallel, the observed suppression of vasomotor-range CBF fluctuations — a physiological oscillatory constrictor component (17) — may be seen as an effective sympathetic withdrawal at the cerebrovascular level, reducing neurogenic vasomotor tone to stabilize flow in a manner similar to decreased heart rate variability during stress (22). At the systemic level, the detected elevations in cortisol, epinephrine, glucagon, and norepinephrine (Figure 1) restore glycemia and sustain systemic perfusion pressure. Nonetheless, the circulating epinephrine has been shown to exert α_1_-adrenergic vasoconstriction, paradoxically reducing microvascular flow (10). However, parenchymal arterioles and capillary pericytes rich in β_2_ receptors have been reported to offset this effect (23, 24), effectively reinforcing the locally initiated metabolic vasodilatation. But most likely, both endocrine response and CBF reflect a multifactorial convergence of response mechanisms whose combined effect yields the detected correlation.

### Effect of T1D on CBF, vasomotor fluctuations, and hormonal response to HG.

Endocrinologically, early and near-universal loss of the HG-induced glucagon secretion and attenuated sympathetic catecholamine output (Figure 1) reduce mobilization of systemic response mechanisms, yet the ability to raise HG-elicited CBF increase remained largely intact (Figure 3A). While this finding may seem contradictory in the light of some (12, 14, 15) but not all previous reports (25, 26), the distinction between NAH and IAH T1D, often standing at the core of previous research, is important here. The categorization of NAH and IAH in T1D is based on a clinical assessment of an individual’s real-life experience with HG that has been routinely used in this field for more than 20 years. In this dataset, NAH and IAH T1D patients exhibited often diverging response patterns from the levels detected in HCs, effectively averaging out. The preserved or even slightly exaggerated CBF response in NAH T1D patients compared with HCs was contrasted against the marked attenuation of this compensatory mechanism in IAH, most notably within insular cortex, thalamus, striatum, and pallidum (Figure 3, B and C, and Supplemental Table 4). However, the near absence of low-frequency CBF fluctuation suppression in both NAH and IAH suggests a general failure of neurogenic vasoregulatory response. Whether due to defective central-vascular coupling in autonomic neuropathy, albeit otherwise subclinical (27), or vascular structure stiffening and pericyte dysfunction arising from advanced glycation end products in chronic hyperglycemia (28), the vasculature’s ability to follow neural inputs may be compromised. Nevertheless, metabolically driven vasodilation pathways appear to remain largely intact, at least in NAH patients, despite the absence of adequate smoothing of flow dynamics resulting from dysregulated sympathetic tone.

### IAH as a qualitative disruption of global response to HG.

IAH constitutes a multilevel habituation to recurrent HG, combining attenuated afferent signaling from glucosensors in the ventromedial hypothalamus and impaired autonomic response to HG (29). In addition to inadequate cortisol surge (Figure 1), IAH was also associated with blunted thalamic and striato-insular HG-induced hyperemia — regions crucial for interoceptive awareness (30). While this might seem to be a linear deterioration of T1D-related pathology — initial loss of neurogenic vasomotor responsiveness followed by the dysfunction of metabolically driven vasodilatation manifested in loss of CBF response — the qualitatively distinct endocrine-CBF coupling disruption (Figure 4C) portrays IAH rather as a distinct maladaptive phenotype.

The exaggerated positive correlations between both cortisol and epinephrine responses and CBF in IAH (Figure 4C), despite attenuated endocrine reactivity in IAH (Figure 1), may reflect maladaptive sensitization of adrenergic or glucocorticoid receptors after chronic counterregulatory overstimulation, a phenomenon previously implicated in β_2_ adrenergic receptor regulation (31). Conversely, this mechanism may lie at the core of a viable strategy of HG awareness restoration (32). The differences in norepinephrine behavior may be attributable to its cerebrovascular actions dominated by α_1_-adrenergic vasoconstriction (9) in the environment of concurrent suppression of glucagon and cortisol responses (Figure 1). Nonetheless, without more comprehensive insights into the biological interplay between hormonal dysregulation and metabolic alterations, such conclusions remain largely speculative.

Several limitations to this study must be considered. First, although the present cohort constitutes one of the largest yet examined with combined PCASL and endocrine phenotyping, subgroup sizes still limit detection of subtle regional effects and fine-grained analyses of the principal hormonal axes considered. Second, 3 variants of M_0_ calibration were utilized over the course of data acquisition. While an extensive quality control (QC) framework was applied, ultimately necessitating the exclusion of the second protocol, residual interrun variance cannot be entirely ruled out. Third, the cross-sectional design precludes causal inference and remains susceptible to a wide range of confounders — from the intrinsic pathophysiological heterogeneity of the T1D cohort to inconspicuous factors such as mere caffeine intake variability (33). Finally, IAH was classified on the basis of subjective questionnaires about participants’ real-world experience with HG. This is the approach generally taken in classifying individuals in HG research (34), but it does not guarantee that all participants with IAH will have blunted hormonal and symptom responses to HG compared with participants with NAH. Events in the weeks before the clamp study, such as HG or hyperglycemia episodes, exercise, and sleep disturbance, have all been hypothesized to impact the response to clamped HG, but these variables were not controlled in our study. In addition, C-peptide concentration assessment was not implemented within the study protocol, thus precluding the evaluation of eventual residual β cell function as the basis for the reported modest glucagon responses in the NAH group.

Future research would benefit from multicenter longitudinal designs following T1D patients over several years, employing the latest PCASL protocols established within Human Connectome Project (HCP) efforts with adequate off-resonance artifact mitigation and participant-specific hematocrit measurements. Ideally, such investigations would incorporate more complex evaluation of hormonal and pancreatic polypeptide responses (35), history of severe HG episodes (36), additional modalities further elucidating CBF fluctuations, and other direct and surrogate markers of autonomic system tone.

### Conclusion.

The present study extends the prevailing understanding of HG-induced regionally targeted CBF increase into a more nuanced and dynamic cerebrovascular response also involving the suppression of autonomic CBF oscillations, all tightly linked to endocrine activation. Moreover, our findings indicate that T1D in general disrupts the dynamic component of cerebrovascular control, whereas IAH, governed by qualitatively altered neuro-endocrine integration, further compromises CBF reserve. These insights underscore not only the heightened cerebral vulnerability associated with recurrent HG, but also the imperative to foreground neurovascular-endocrine models in diabetes research.

## Methods

### Sex as a biological variable.

Group allocation was based on HG awareness status, irrespective of sex. Sex was included as a nuisance variable in all statistical analyses to account for potential sex-related differences. The findings are therefore expected to be relevant to both sexes, even though the study was not intended, nor powered, to test for sex-specific effects.

### Participants.

Seventy-nine patients with T1D (HbA1c < 8 %) were recruited from the Endocrine Clinic at the University of Minnesota between 2015 and 2020. Forty-five age-, sex-, and BMI-matched healthy volunteers (HCs) were enrolled from the local community. Exclusion criteria comprised history of stroke, seizures, neurosurgical procedure, cardiac arrhythmia, pregnancy, or medication altering glucose homeostasis (with the exception of insulin). IAH was defined by a Clarke score > 3 (37) (or a Clarke score = 3 with a Gold score > 3) (38); scores below these thresholds were considered NAH.

### Experimental protocol.

Studies were conducted in the morning, typically beginning around 9 am. After an overnight fast, 2 intravenous cannulae were inserted (forearm for infusions and lower leg vein for sampling following arterialization of the venous blood as previously described in ref. 39), and the ambulatory insulin pump, if present, was removed. Variable insulin infusion was titrated to achieve euglycemia (EU) (BG ≈ 95 mg/dL), guided by BG measurements performed every 5 min (Analox). At time 0, the insulin rate was fixed at 2.0 mU/kg/min and potassium phosphate (4 mmol/h) was initiated. Stable BG at 95 mg/dL was maintained via a variable 20% dextrose infusion during the anatomical and PCASL scans (~25 min). The dextrose infusion was then temporarily stopped, allowing BG to fall to 50 mg/dL; this HG plateau was held for approximately 20 min using variable 20% dextrose infusion during a further PCASL acquisition before BG was restored to EU. Two baseline and 3 HG samples were stored for glucagon, epinephrine, norepinephrine, and cortisol assays. Symptom scores (0–6 for 12 autonomic and neuroglycopenic symptoms) (40) were recorded immediately before scanning (EU) and at the end of the HG plateau. Upon completion, infusions were discontinued, a meal was provided, and T1D participants resumed their usual insulin regimen.

A schematic of the clamp sequence is provided in Supplemental Figure 1.

### MRI protocol.

Scanning was performed on a Siemens 3T MAGNETOM Prisma with the following sequences: (a) T_1_-weighted MPRAGE: 1 mm isotropic; repetition time (TR)/echo time (TE)/inversion time = 2,150/2.47/1,000 ms; flip angle 8°; GRAPPA 2; (b) T_2_-weighted SPACE: 1 mm isotropic; TR/TE = 3,200/147 ms; GRAPPA 2; and (c) PCASL: TR/TE = 5,000/14 ms; 36 slices, 3 mm isotropic, 20% interslice gap, 2D single-shot gradient-echo echo-planar imaging readout; field of view 210 × 210 mm². The following parameters were employed: labeling duration = 1,600 ms, single postlabeling delay = 1,600 ms; labeling slab 10 mm thick, 90 mm caudal to the center of the imaging volume; and 80 label/control pairs per run (41). For equilibrium magnetization (M_0_) calibration, 3 calibration variants were used over the duration of the study: 1 M_0_ image was acquired before the label control series, 1 M_0_ image was acquired after the series, and 2 M_0_ images were acquired after the series.

PCASL was acquired separately during EU, EU→HG transition (excluded from subsequent analyses), and HG.

### Neuroimaging data preprocessing.

PCASL data were preprocessed using a custom pipeline built around the Bayesian Inference for Arterial Spin Labelling framework (42) and integrated with the HCP minimal preprocessing pipeline (43). After rigid-body motion correction, the analysis used a single-compartment kinetic model with a fixed bolus duration and a single postlabeling delay, employing individualized arterial blood T_1_ based on age- and sex-related hematocrit values derived from predefined age bins (44, 45). Absolute quantification used M_0_-based calibration referenced to cerebrospinal fluid (CSF), incorporating TE-specific T_2_ correction for CSF and arterial blood and reference CSF T_1_/T_2_ (tissue relaxation parameters were used exclusively for calibration, not the kinetic model). If 2 M_0_ images were present, their average was used. A Bayesian inference framework with spatial regularization imposed by Gaussian Markov random field prior was applied. Posterior CBF maps were deconvolved with the resampled tissue probability maps derived from the subject’s structural image to separate perfusion signal contributions from gray and white matter. The final outputs included absolute CBF maps and calibrated perfusion time series based on a sliding window approach, whereby each epoch consisted of 2 consecutive label-control pairs with an overlap of 1 adjacent label-control pair (i.e., 39 partially overlapping epochs per each dataset). This strategy allowed for reasonably fine-grained temporal characterization of perfusion dynamics while preserving sufficient signal averaging within each window to maintain robustness to noise.

Both mean CBF maps (estimated from the full acquisitions) and CBF time series were projected onto subject-specific mid-thickness cortical surfaces, with mapping constrained to the cortical ribbon derived from the HCP minimal preprocessing pipeline. Interpolation was performed along the cortical thickness direction, weighting voxel contributions according to the proportion of gray matter intersected by the sampling trajectory to reduce partial volume effects from adjacent white matter and CSF. A multimodal HCP atlas (46) was used for subsequent parcellation, providing 180 cortical parcels per hemisphere. Subcortical segmentation was based on multiple probabilistic atlases collectively covering all subcortical gray matter structures: Oxford thalamic connectivity atlas (47), Oxford-GSK-Imanova connectivity striatal atlas (48), probabilistic structural cerebellar atlas (49), ATAG atlas (50), hypothalamus atlas (51), and ARAS atlas (52). Furthermore, high-resolution FreeSurfer automatic subregion segmentation for hippocampus substructures and amygdala nuclei was implemented (53, 54). Relevant masks were nonlinearly warped from standard atlas space to each subject’s native T_1_-weighted anatomical space (standard space atlases) and rigid-body transformed to the target PCASL space in 1 step (both standard space atlases and FreeSurfer-derived individual hippocampus and amygdala subfields). For each region of interest, the weighted average signal was computed as the ratio of the voxel-wise intensity-probability product to the sum of probabilities to ensure each voxel’s contribution was scaled by its probabilistic membership in the respective region.

For each subject, CBF time series parcellated in this manner was linearly detrended and truncated to an even length before applying a fast Fourier transform. Power spectral density was derived from the squared fast Fourier transform amplitude, and CBF oscillation amplitude was defined as the square root of the total power within the neurogenic vasomotor 0.02–0.05 Hz frequency band (17–19).

In addition to visual review of both raw and processed PCASL data, quantitative QC was implemented at 3 tiers. First, hard veto excluded any PCASL acquisition (EU or HG) exhibiting excessive motion spikes (95th percentile framewise displacement > ½ voxel width [1.5 mm]). Second, each acquisition (EU and HY) was subjected to acquisition-wise multivariate QC based on 5 reasonably orthogonal stability metrics: head motion (95th percentile framewise displacement), sudden intensity jumps (DVARS of tag–control difference series), temporal stability (temporal signal-to-noise ratio of tag–control difference), low-frequency hardware drift (power spectral density integrated over 0–0.01 Hz), and distributional shape of mean CBF map (skewness). Robust Mahalanobis distances were computed from the orthogonalized Gnanadesikan–Kettenring scatter matrix. Acquisitions with *D*^2^ > χ^2^(5; 0.99) = 15.09 were deemed outliers and relayed for the next level of QC. Third, plausibility check converted parcellated mean CBF, vasomotor band power spectral density (0.02–0.05 Hz), and global connectivity strength to robust *z* scores within each condition; |*z*|> 3 disqualified acquisitions previously flagged by tier 2. Only participants whose both condition acquisitions satisfied these criteria were retained.

### Counterregulatory response modeling.

Singular missing hormone measurements (0.3% of all data points) were imputed using modified Akima piecewise interpolation within each participant’s hormone time series to preserve local monotonicity and minimize artifactual oscillations. Residual gaps (an entire hormone panel missing for 5 participants, 1.5% of data points) were substituted with group- and hormone-stratified time point means. All subsequent tests were conducted on the imputed data set.

Before modeling the cerebral-perfusion response to HG and its coupling with the counterregulatory response to address our aims, we wanted to understand the group-specific counterregulatory responses to HG. Hormonal responses were analyzed using LMEM to account for interindividual variability in both baseline levels and sampling times. Each hormone was modeled independently, with time relative to HG onset (2 consecutive BG measurements < 50 mg/dL) entered as a continuous fixed-effect predictor and a random intercept specified for each participant to account for intra-individual correlation in repeated measures. Degrees of freedom were estimated using Satterthwaite approximation. Group comparisons in hormonal linear trajectories were tested for (a) HCs versus pooled T1D patients and (b) T1D IAH versus NAH separately. To assess the multivariate (global) intergroup divergence in hormonal profiles, a nonparametric permutational multivariate analysis of variance (PERMANOVA) was conducted on subject-wise trajectories using Mahalanobis distance to account for interhormonal covariance, with 10,000 permutations per test. Comparisons were performed separately for HCs versus pooled T1D patients and IAH versus NAH. All reported *P* values across hormones and model terms of interest (LMEM and PERMANOVA jointly) were corrected using the Benjamini-Hochberg FDR procedure.

### Statistics.

Absolute CBF and CBF fluctuation differences were considered in the analysis to preserve consistency with standard hormonal response evaluation and prior literature, as a physiologically meaningful, directly interpretable quantity (mL/100 g/min for CBF) and to avoid potential baseline-dependent bias. All presented analyses were performed on the HG–EU differences rather than on the separate EU or HG condition.

Changes in CBF and its low-frequency fluctuations in response to HG, as well modulations of the HG effect by hormonal responses, were analyzed using nonparametric permutation testing in the PALM toolbox (55). All analyses were conducted on parcellated CIFTI maps with 10,000 permutations per test and anatomical proximity-based threshold-free cluster enhancement, followed by distribution tail approximation. Correction for multiple testing was applied using FDR across parcels, modalities (CBF and its low-frequency fluctuations), and contrasts of interest, with a statistical significance threshold of α = 0.05. Designs involving multiple hormonal predictors employed constrained maximum statistic combination to restrict inference to the contrasts relevant to the interaction term.

The following GLMs, all including age and sex as nuisance covariates, were tested.

(a) Canonical physiological neurovascular response to HG and coupling with hormonal response: within-subject contrast of HG versus EU and interaction analysis of that contrast with separate regressors for each hormonal response, including an omnibus *F* test for joint evaluation encompassing all hormones. Both GLMs were limited to HCs.

(b) Distortion of the neurovascular response to HG: between-group contrast comparing the main HG versus EU effect for HCs versus pooled T1D cohort and separately for IAH versus NAH T1D. As a sensitivity analysis, a supplemental 3-group, 1-way ANOVA (HC, NAH, and IAH) was performed with a pooled variance across all 3 groups and to evaluate robustness to the alternative grouping scheme.

(c) Distortion of hormonal-neurovascular coupling: assessed by regressing neurovascular response on 4 hormone responses, groups, and their interactions. A multivariate *F* test evaluated whether hormone-neurovascular response slopes differed between groups, separately for CBF and for its low-frequency fluctuations as neurovascular metrics, and separately for the HC versus pooled T1D cohort and IAH versus NAH T1D as groups.

### Study approval.

All procedures conformed to the Declaration of Helsinki and the US Code of Federal Regulations and were approved by the Institutional Review Board of the University of Minnesota (1608M92941 and 0301M40641). Written informed consent was obtained from all participants before the study.

### Data availability.

Unprocessed datasets are not publicly available due to the sensitive nature of the dataset. However, data will be provided upon reasonable request to the corresponding author. Processed parcellated outputs are provided in the supplemental tables. Values for all data points in graphs are provided in the Supporting Data Values file.

## Author contributions

Conceptualization: PF, LEE, ERS, and SM. Data curation: PF, AC, HG, AFK, and SM. Formal analysis: PF, YZ, LEE, and SM. Funding acquisition: SM. Investigation: PF, AC, HG, AAM, AFK, and SM. Methodology: PF, XL, ERS, and SM. Software: PF and AC. Visualization and writing (original): PF and SM. Writing (review and editing): PF, AC, HG, AAM, AFK, XL, YZ, LEE, ERS, and SM.

## Conflict of interest

The authors have declared that no conflict of interest exists.

## Funding support

This work is the result of NIH funding, in whole or in part, and is subject to the NIH Public Access Policy. Through acceptance of this federal funding, the NIH has been given a right to make the work publicly available in PubMed Central.

- NIH awards P41-EB-015894, P30-NS-076408, R01-DK-099137 (to SM), R56-DK-099137 (to SM), and DP1 AG093028 (to SM).
- National Center for Advancing Translational Sciences of the NIH (awards KL2-TR-000113 and UL1-TR-000114).
- Charles University, Czech Republic (Cooperatio Program, research area NEUR), Brain Dynamics (grant number CZ.02.01.01/00/22_008/0004643) to PF.
- General University Hospital in Prague (MH CZ-DRO-VFN64165) to PF.

## Supplementary Material

Supplemental data

ICMJE disclosure forms

Supplemental table 1

Supporting data values

## Figures and Tables

**Table 1:**
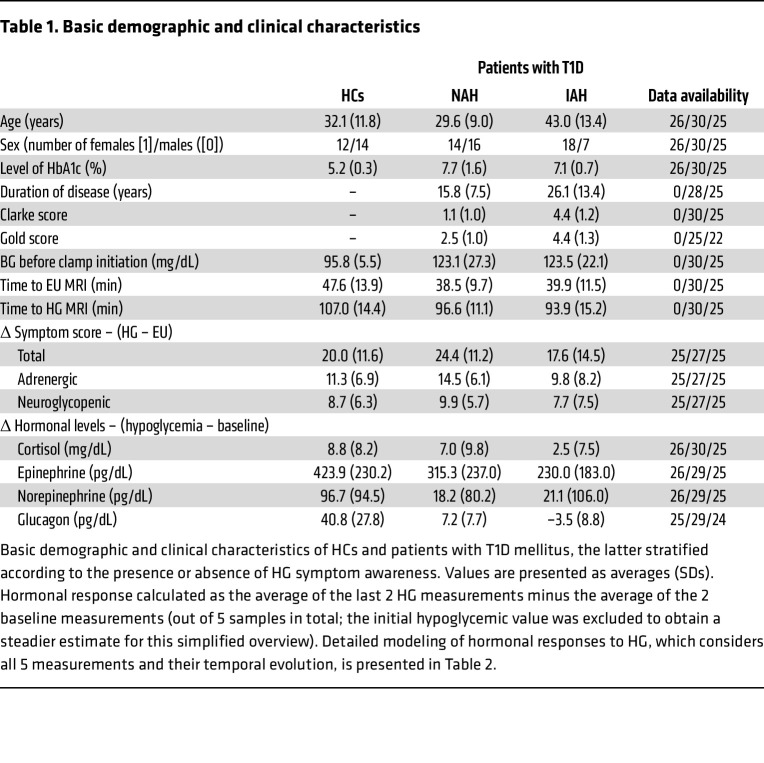
Basic demographic and clinical characteristics

**Table 2:**
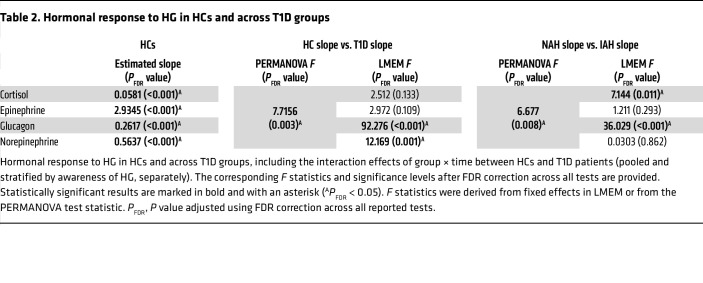
Hormonal response to HG in HCs and across T1D groups

## Version 1 02/19/2026

In-Press Preview

## Version 2 04/15/2026

Electronic publication
