# Supplementary material for: Disrupted neurovascular-endocrine coupling in type 1 diabetes with impaired awareness of hypoglycemia
Source: J Clin Invest. 2026 Feb 19;136(8):e199725. doi: 10.1172/JCI199725 (PMC13078881; doi:10.1172/JCI199725)

Supplemental Figure 1: Experimental hypoglycemia (HG) session. After a 30 min fasting baseline, blood glucose (BG) level was clamped at 95 mg/dL, lowered to 50 mg/dL for ≈20 min, and then returned to 95 mg dL. Grey shading marks the period of BG adjustments. The black bars show the continuous pseudo-continuous arterial spin-labeling (PCASL) MRI acquisitions; grey bar immediately beforehand indicates the acquisition of anatomical scans. Red crosses indicate BG sample collection performed every 5 minutes. Two baseline and three HG blood draws (grey boxes labelled CR) were used for counterregulatory hormone assays.

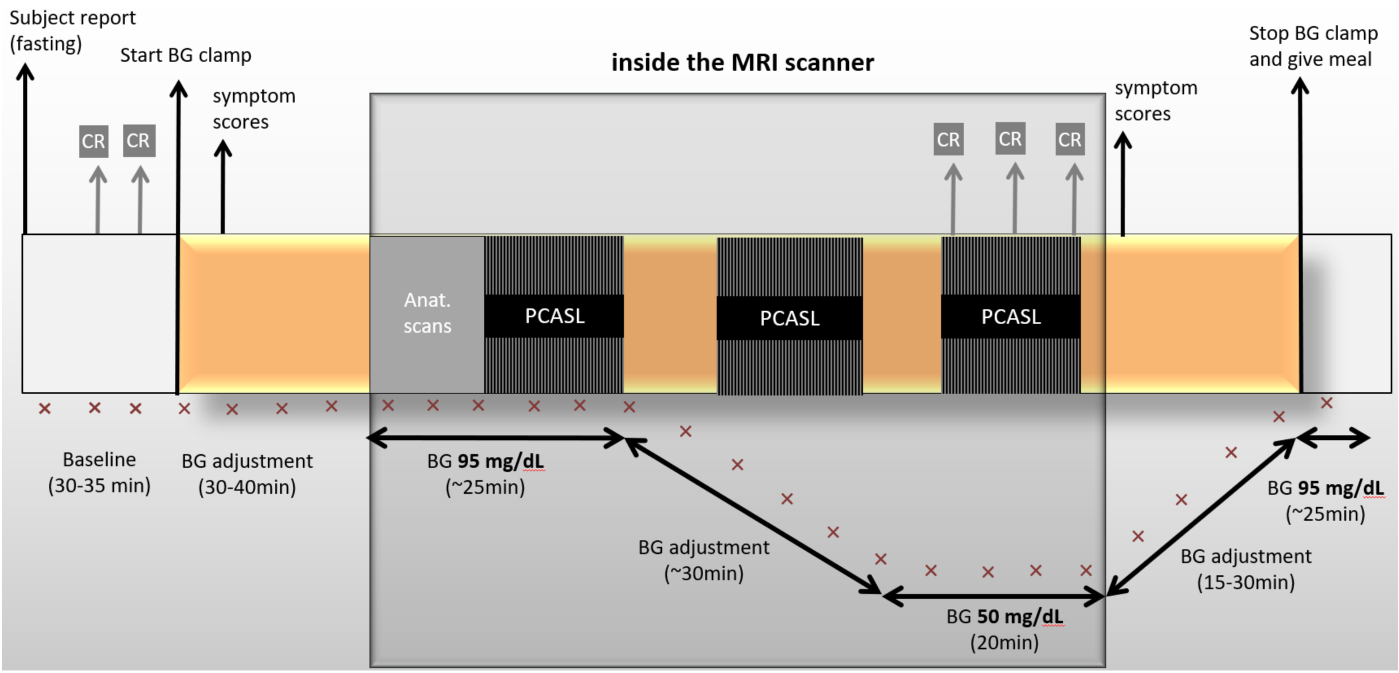

Supplemental Figure 2: Flowchart of participant screening and data exclusion. Abbreviations: IV – Intravenous; MRI – Magnetic Resonance Imaging; PCASL – Pseudo-continuous Arterial Spin Labeling; eu - euglycemia. Incompatible PCASL protocol refers to the second acquisition scheme with one M_0 volume at the end of full tag-control series, which exhibited two orders of magnitude larger robust Mahalanobis distance (median $D^2 \approx 525$) than the other two acquisition protocols (median $D^2 \approx 5.5$), prompting the exclusion of all datasets acquired within this scheme from the analysis.

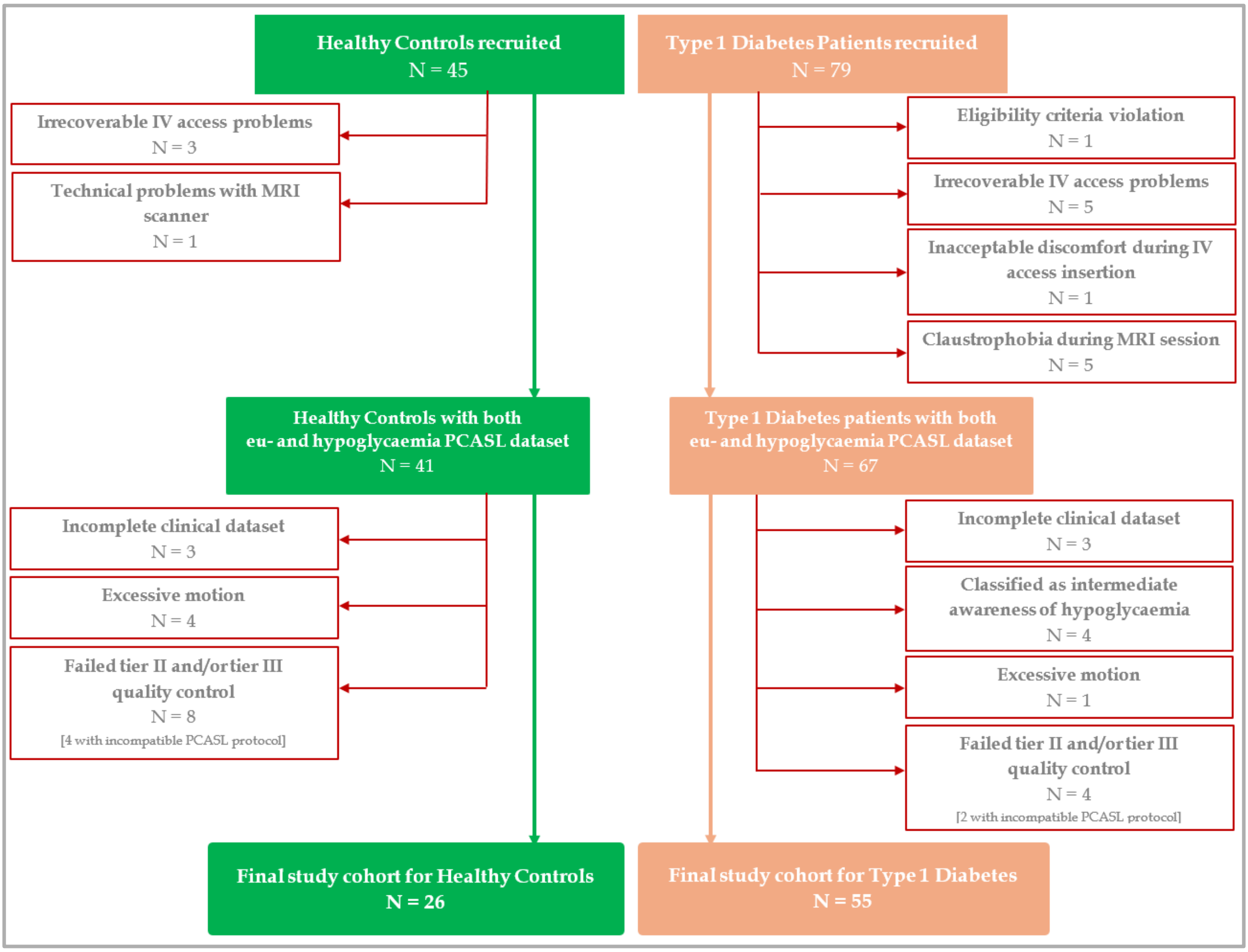

Supplemental Figure 3: Lasagna plot illustrating individual blood glucose trajectories during hypoglycemic clamp across all participants – stratification into healthy controls, type 1 diabetes patients with normal awareness of hypoglycemia and impaired awareness of hypoglycemia. Each row represents a single participant; columns depict measured blood glucose value (absolute concentration in mg/dL) with 5-minute sampling interval; zero timepoint (also marked by dashed line) corresponds to the initiation of hypoglycemic clamp. Colours reflect absolute blood glucose values according to the scale on the right.

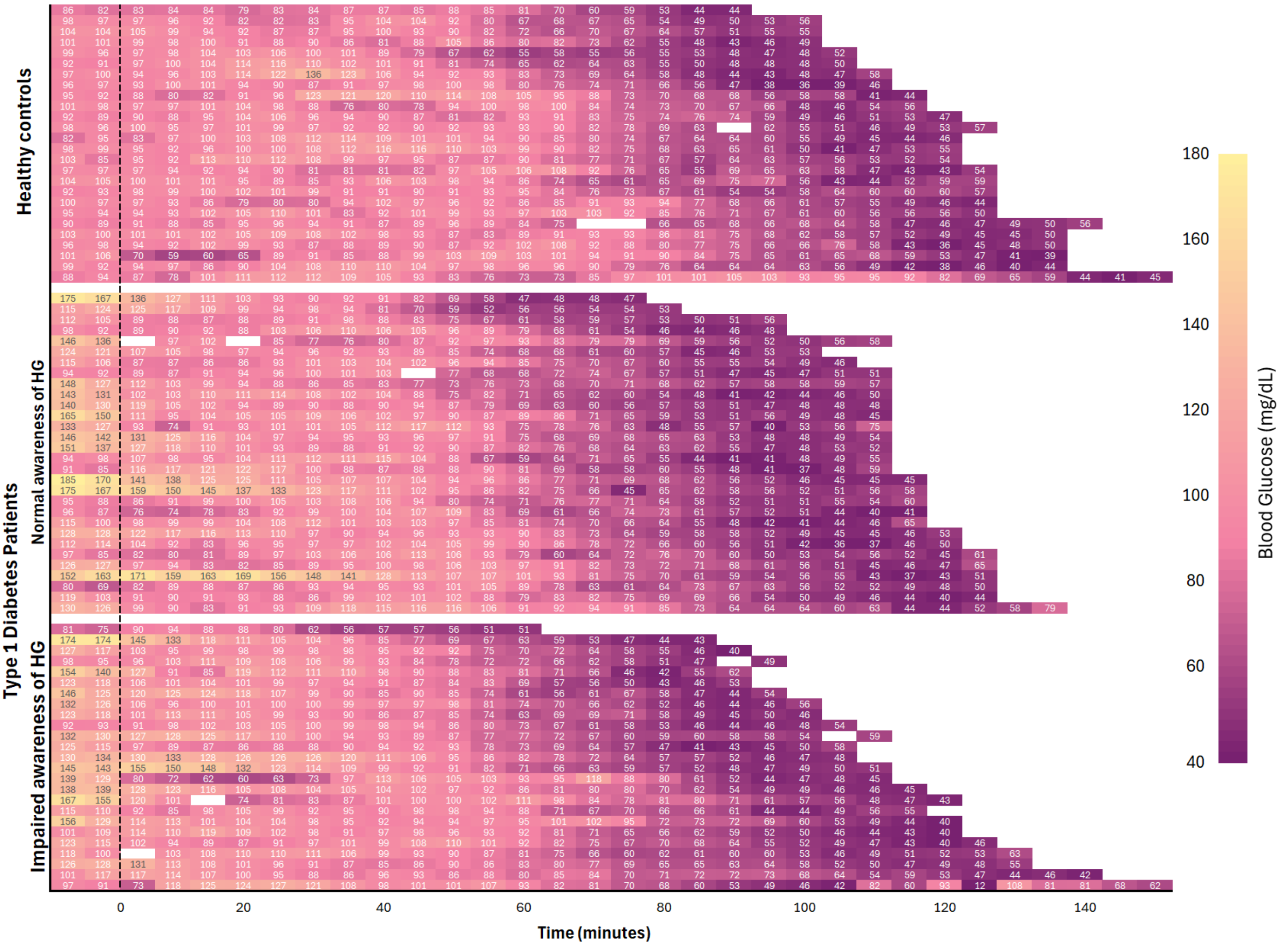

Supplemental Figure 4: Objective symptom response to hypoglycemia. Bar heights above 0 represent subgroup averages of individual hypoglycemia–euglycemia difference in total Symptom Score (or its adrenergic and neuroglycopenic subcomponents). Individual participant values depicted as grey circles, with jitter along the x axis for better visibility. No statistically significant inter-group differences detected. See Table 1 for further information. Abbreviations: HC – Healthy Controls; T1D – Type 1 Diabetes; NAH – T1D with Normal Awareness of Hypoglycemia; IAH – T1D with Impaired Awareness of Hypoglycemia.

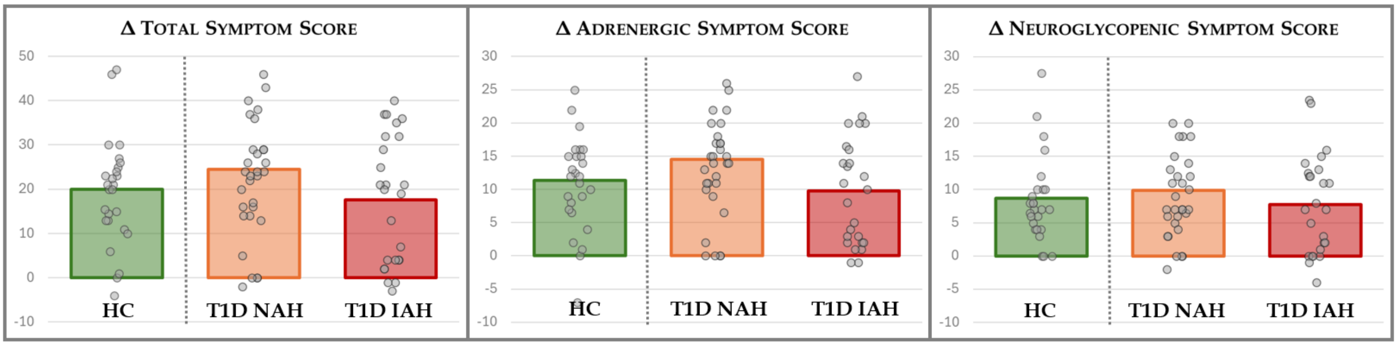

Supplemental Figure 5: Sensitivity analysis: three-group ANOVA (healthy controls – type 1 diabetes patients with normal awareness of hypoglycemia – type 1 diabetes patients with impaired awareness of hypoglycemia) for cerebral haemodynamic response to hypoglycemia, shown separately for Cerebral Blood Flow and Spontaneous Cerebral Blood Flow Oscillations. Values correspond to -log(p) with threshold-free cluster enhancement, thresholded at 1.3 (i.e. $p<0.05$), False Discovery Rate corrected jointly across parcellation units and modalities. Subcortical effects illustrated on six axial slices (MNI z = 15, 6, -3, -12, -21, -30). Images follow neurological convention (right = right).

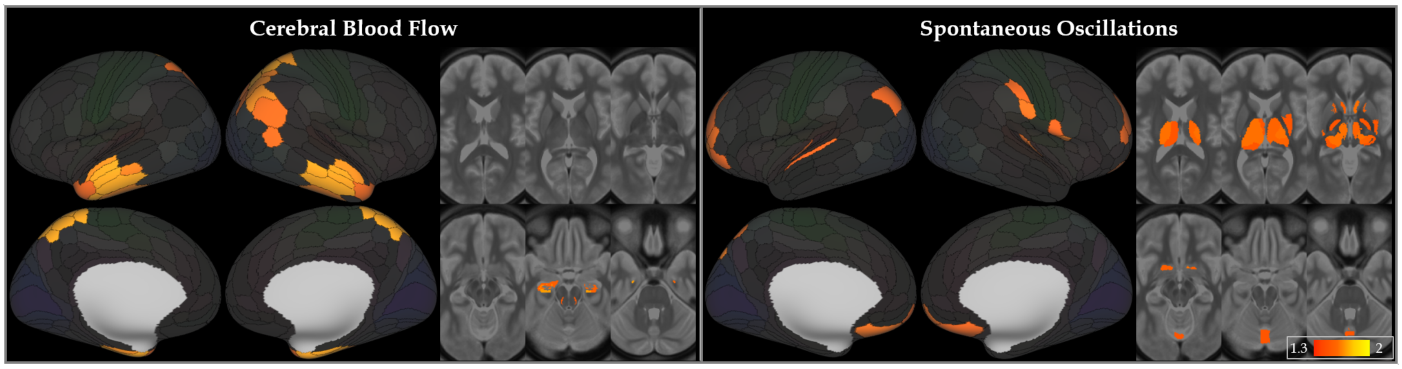

Supplement: Supplemental data [file jci-136-199725-s202.pdf]
